# Acute skeletal muscle wasting and dysfunction predict physical disability at hospital discharge in patients with critical illness

**DOI:** 10.1186/s13054-020-03355-x

**Published:** 2020-11-04

**Authors:** Kirby P. Mayer, Melissa L. Thompson Bastin, Ashley A. Montgomery-Yates, Amy M. Pastva, Esther E. Dupont-Versteegden, Selina M. Parry, Peter E. Morris

**Affiliations:** 1 Department of Physical Therapy, College of Health Sciences, University of Kentucky, 900 Rose St, Wethington 204D, Lexington, KY 40536 USA; 2 Center for Muscle Biology, University of Kentucky, Lexington, USA; 3 College of Pharmacy, University of Kentucky, Lexington, USA; 4 Division of Pulmonary, Critical Care and Sleep Medicine, College of Medicine, University of Kentucky, Lexington, USA; 5 Departments of Orthopedic Surgery, Medicine, Cell Biology, and Population Health Sciences, Duke University School of Medicine, Durham, USA; 6 Department of Physiotherapy, School of Health Sciences, The University of Melbourne, Melbourne, Australia

**Keywords:** Critical illness, Muscle wasting, ICU-acquired weakness, Physical function, Acute respiratory failure, Sepsis, Muscle atrophy, Muscle power

## Abstract

**Background:**

Patients surviving critical illness develop muscle weakness and impairments in physical function; however, the relationship between early skeletal muscle alterations and physical function at hospital discharge remains unclear. The primary purpose of this study was to determine whether changes in muscle size, strength and power assessed in the intensive care unit (ICU) predict physical function at hospital discharge.

**Methods:**

Study design is a single-center, prospective, observational study in patients admitted to the medicine or cardiothoracic ICU with diagnosis of sepsis or acute respiratory failure. Rectus femoris (RF) and tibialis anterior (TA) muscle ultrasound images were obtained day one of ICU admission, repeated serially and assessed for muscle cross-sectional area (CSA), layer thickness (mT) and echointensity (EI). Muscle strength, as measured by Medical Research Council-sum score, and muscle power (lower-extremity leg press) were assessed prior to ICU discharge. Physical function was assessed with performance on 5-times sit-to-stand (5STS) at hospital discharge.

**Results:**

Forty-one patients with median age of 61 years (IQR 55–68), 56% male and sequential organ failure assessment score of 8.1 ± 4.8 were enrolled. RF muscle CSA decreased significantly a median percent change of 18.5% from day 1 to 7 (*F* = 26.6, *p* = 0.0253). RF EI increased at a mean percent change of 10.5 ± 21% in the first 7 days (*F* = 3.28, *p* = 0.081). At hospital discharge 25.7% of patients (9/35) met criteria for ICU-acquired weakness. Change in RF EI in first 7 days of ICU admission and muscle power measured prior to ICU were strong predictors of ICU-AW at hospital discharge (AUC = 0.912). Muscle power at ICU discharge, age and ICU length of stay were predictive of performance on 5STS at hospital discharge.

**Conclusion:**

ICU-assessed muscle alterations, specifically RF EI and muscle power, are predictors of diagnosis of ICU-AW and physical function assessed by 5x-STS at hospital discharge in patients surviving critical illness.

## Background

Patients surviving critical illness have significant skeletal muscle wasting and dysfunction, including weakness and atrophy [1, 2]. Up to two-thirds of patients admitted for critical illness will be diagnosed with intensive care unit-acquired weakness (ICU-AW) [3], leading to deficits in physical function [4, 5]. As a result, survivors have long-term physical disability leading to difficulty performing activities of daily living (ADL), such as standing up from a chair, and deficits in these basic ADLs are highly associated with poor health related-quality of life (HRQoL) [6, 7]. Observational and single-center randomized controlled trials demonstrate that physical rehabilitation provided in the ICU may positively influence short- and long-term patient outcomes, including greater muscle strength at ICU discharge, reduced mechanical ventilation duration and improved HRQoL [8–11]. Moreover, clinical practice guidelines recommend physical rehabilitation for mitigating the detrimental effects of immobilization that occur during critical illness [12, 13]. However, recent ICU-based physical rehabilitation randomized controlled trials (RCTs) fail to demonstrate robust immediate or long-term functional benefits [11, 14–18]. One potential explanation for these results is subject heterogeneity [19]. Also, rehabilitation trials rarely implement or stratify interventions based on muscular dysfunction leading to “one-size fits-all” interventions.

Muscle strength and muscle power are vital components of muscular function. Muscle power is differentiated from muscle strength in that power accounts for velocity (distance/time) of force production, while muscle strength is the ability to generate maximal muscle force only [20]. Muscle power is crucial for daily activities that require velocity to overcome distance or gravity, such as standing up from a chair or from a toilet [21, 22]. However, muscle power is not a current focus in ICU or hospital rehabilitation. Assessment of muscle power is novel in this population, and deficits in power suffered during critical illness may help explain persistence of physical function impairments.

Early classification of muscle wasting and dysfunction, including the degree of deficit, is important for appropriate allocation of rehabilitation interventions, but difficult due to the heterogeneity and severity of acute critical illness [23]. Muscle ultrasound has gained significant traction as a tool to assess and track changes in skeletal muscle potentially improving classification of patients who may be at risk for muscular or physical impairments. However, data surrounding muscle ultrasound are conflicting. A recent study demonstrated that muscle size measured at day 7 of ICU admission was not predictive of ICU-AW [24], while an observational study in a cohort of 22 critically ill patients demonstrated that muscle size and quality were associated with physical function at ICU discharge [25]. Additionally, earlier and greater change in muscle size measured by ultrasound was associated with in-hospital mortality, mechanical ventilation (MV) duration and ICU-AW [26]. Conflicting evidence may be attributed to the heterogeneity in patient populations and potentially discrepancies in user approach leading to variations in practice and human operator error [27]. Currently, there is a need to determine whether muscle mass, quality and function assessed in ICU are related to or predictive ICU-AW and physical function at hospital discharge. The purpose of this study was to determine whether muscle alterations assessed during an ICU stay by changes in muscle size, quality, strength and power, are associated with or predict diagnosis of ICU-AW and physical function at hospital discharge.

## Methods

### Ethical considerations

This study was reported in accordance with the Strengthening the Reporting of Observational Studies in Epidemiology (STROBE) guidelines and approved by the Institutional Review Board at the University of Kentucky. Research subjects or legally authorized representative provided written informed consent before participating in the study. Consent was obtained from a legally authorized representative for patients unable to give consent due to sedation, mentation and/or consciousness, and re-consent was obtained once patient was awake, stable and could provide informed consent themselves.

### Study design

A prospective, longitudinal observational study was conducted with adult patients admitted to Medicine ICU (MICU) or the Cardiothoracic ICU (CTICU) and enrollment occurred from November 15, 2018, to July 15, 2019. Eligibility criteria were: 18 years of age or older with a primary or secondary diagnosis of acute respiratory failure (ARF) or sepsis of any origin that were anticipated to spend more than 3 days in the MICU/CTICU and survive the current hospitalization and enrolled within 48 h of admission. In 2019, patients admitted to MICU had a variety of admitting diagnoses with a mean sequential organ failure assessment (SOFA) of 6.3 with mean ICU length of stay (LOS) of 4.9 days and all-cause mortality of 21% [28]. Patients in the CTICU have a similar acuity level requiring critical care for postoperative cardiac and thoracic surgery, as well as patients requiring extra-corporeal membrane oxygenation for any indication. Thus, the inclusion criterion with diagnosis of ARF and sepsis was utilized to set a minimum severity level to reduce the heterogeneity given the MICU and CTICU have a diverse patient population with range of severity of illness. Patients were excluded from enrollment if they had baseline cognitive impairments, were non-ambulatory prior to hospitalization, had a pre-existing neurologic or neuromuscular disorder, new traumatic injury with lower-extremity fracture, one or more amputations of lower-extremity, were pregnant, admitted for substance abuse or were otherwise inappropriate for study procedures as determined by the primary attending physician. Patients with morbid obesity (body mass index (BMI) > 45 kg/m^2^) were excluded to reduce distortion of ultrasound images.

### Study procedures

#### Muscle ultrasound

The right quadriceps femoris muscle and the right tibialis anterior (TA) were assessed for muscle size and echointensity (EI) with the SonoSite IViz (FUJIFILM SonoSite Inc. Bothell, WA) portable ultrasound with 8.5-MHz linear transducer on ICU days 1, 3, 5 and 7. Ultrasound device settings were kept constant for subjects across time points with the same sonographer (KM, physical therapist, PhD, > 4 years of muscle ultrasound experience) acquiring all images [29]. The methods for image acquisition and analysis of quadriceps and TA were previously reported [1, 30] and have good to excellent reliability [29, 31–33]. Minimal probe compression and depth of 5.9 cm were utilized to obtain three images at all time points of both muscles. Quadriceps femoris muscle imaged at 2/3 distance from anterior superior iliac spine (ASIS) to superior patella border and TA muscle imaged at 1/3 distance from lateral tibial plateau to inferior border of the lateral malleolus. Images were saved on the device hard drive and transferred to computer for analysis using ImageJ software (NIH, Bethesda, MD). The average value of three consecutive images was used in analyses [25, 27]. Quadriceps femoris ultrasound images were analyzed for quantification of rectus femoris (RF) muscle cross-sectional area (CSA), RF muscle thickness (mT), quadriceps complex (QC) muscle thickness (rectus femoris plus vastus intermedius thickness) and for muscle quality (EI) [29]. TA muscle ultrasound images were analyzed for mT, CSA and EI. The final analyses included two approaches: CSA, mT and EI on ICU day one of admission to ICU (baseline) and parameters as percentage change from ICU day 1 to day 7.

Prior to volitional assessments, the patient had to be oriented (determined as ability to complete 3 of 4 domains of name, birthday, location and date) and follow simple commands by scoring ≥ 3/5 on DeJonghe criteria [34].

### Muscular strength, power and physical function

*Muscle strength* was assessed using three different techniques at ICU discharge and hospital discharge:

1. The Medical Research Council-sum score (MRC-ss) is a measure of global peripheral limb muscle strength that is standard of care for diagnosing ICU-AW with less than 48/60 denoting diagnosis [34–37].
2. Muscle strength force production and the rate of force development of the right knee extensors and right ankle dorsiflexors were recorded using a handheld dynamometry (HHD) (Lafayette Manual Muscle Test System Model-01165, Lafayette Company, Lafayette, IN) [38]. HHD to assess isometric muscle strength is reliable and correlated with the gold standard of isokinetic dynamometry [38]. Knee extension was measured in supine or semi-reclined (head of bed < 30 degrees) position with 20 degrees of knee flexion using a roll with dynamometer positioned proximal to the foot on the tibia [39]. Ankle dorsiflexion was measured with the knee in ~ 5 degrees of flexion (small towel under the knee) and supported on a hospital bed or leg rest with the ankle in neutral with dynamometer positioned on the dorsum of the mid-foot. Patients unable to extend lower limb or dorsiflexion foot against gravity (< 3/5 on MRC-ss for knee extension and ankle dorsiflexion) did not perform HHD. Patients participated in a minimum one practice repetition with therapist providing standardized verbal cues for activation, direction and encouragement. The peak value of six second contraction was recorded, and the average of three repetitions was used in analyses with patients resting a minimum of 30 s between repetitions.
3. Handgrip strength of dominant hand was assessed at ICU discharge and hospital discharge using the Jamar hydraulic dynamometer (Sammons Preston Rolyan, Bolingbrook, IL, USA) with technique, position and cues previously described [37, 40]. The average of the peak values for three repetitions was utilized in the analysis.

- *Muscle power* was assessed at or prior to ICU discharge and again at hospital discharge with a linear potentiometer (HUMAC-360, CSMi, Stoughton, MA) to record the velocity and peak-velocity of a unilateral lower-extremity press using a Shuttle MiniPress (Shuttle Systems, Bellingham, WA) while sitting in hospital bed or seated in hospital chair [41]. Subjects performed three repetitions of the leg press at two pre-determined levels of resistance, 2 lbs and 10% of bodyweight. Patients were permitted to perform three repetitions for familiarization prior to formal testing.
- *Physical functional outcomes* The primary physical function outcome of interest was performance of 5-times sit-to-stand test (5 × STS) at hospital discharge since it is a fundamental component of mobility and an independent measure of muscle strength and power [42]. The short performance physical battery (SPPB) [43, 44], six-minute walk distance (6MWD) [45, 46] and clinical frailty scale (CFS) were assessed at hospital discharge. The CFS is validated tool assessing frailty based on mobility status, cognitive and physical function, and levels of independence [47].
- *Standard rehabilitation and nutrition care* Patients admitted to MICU/CTICU receive physical therapy and occupational therapy as standard of care initiated by order at the discretion of the primary attending. Physical and occupational therapy sessions typically occur 2–5 times per week lasting ~ 30 min and initiated upon weaning of sedation with MICU and CTICU medical teams attempting to follow the ICU Liberation Bundle (A-F) [13]. Patients requiring sedatives and not appropriate for active mobilization receive passive range of motion at minimum three times delivered daily by a mobility technician or nursing staff. Active mobilization is initiated by the interdisciplinary team as soon as sedation is weaned and hemodynamic stability is reached per prior recommendations [48]. The Physical Function in the ICU Test (PFIT-s) was performed by staff physical therapists according to routine care which includes performing the test upon initial evaluation in the ICU [49, 50]. Nutritional practice in our institution aligns with the SCCM/ASPEN guidelines for critically ill adults [51]. Our nutrition support service assesses all ICU patients and provides an individualized enteral nutrition plan within 24 to 48 h of ICU admission for patients without volitional intake. Enteral and volitional daily nutritional goals are based on 25 kilocal/kilogram per day for caloric intake (kilocal) and 1.2–2.5 g/kilogram per day of protein [51].
- *Clinical variables* Baseline demographics (age, sex, BMI), Charlson comorbidity index (CCI) and critical illness data including ICU admission diagnosis, sequential organ failure assessment (SOFA), hours of mechanical ventilation (MV), ICU and hospital length of stay (LOS), time to first rehabilitation session, number of rehabilitation sessions, sedation (yes/no), use of inotropes and vasopressors (yes/no) and mortality (defined as in-hospital mortality plus transfer to inpatient hospice) were assessed.

### Statistical considerations

*Sample size* A priori sample size calculation was not performed. The sample size was pragmatically based on 8-month time frame as well as previously published literature [1, 25].

### Statistical analysis

Data were assessed using descriptive statistics including mean and standard deviation (SD) or median and interquartile range (IQR), histograms and Shapiro–Wilk test for normality. Ultrasound data were examined for change over time using a linear mixed-model approach. The relationships between muscle ultrasound parameters, muscle power, muscle strength, demographics, clinical and physical function data were assessed with Spearman Rho tests. A multivariate logistic regression model was created to assess the effects of independent variables on development of ICU-AW at hospital discharge. Variables identified for the model included baseline demographics (age, sex, BMI) and other variables that are purported to be associated with weakness including muscle size and quality, severity of illness, ICU length of stay and muscle power. Stepwise backward regression at the 0.2 level was used to minimize overfitting. Power assessment (10% BW) at ICU discharge was forced into the model, as this is our primary exploratory predictor variable. Using the same approach, a multivariate linear regression was used to assess the relationship between predictor variables with dependent variable of 5-times sit-to-stand performance at hospital discharge. The models were tested for assumptions of logistic and linear regression as appropriate. Multicollinearity was assessed using variance inflation factor; normality of errors was assessed with the IQR test. We assessed model fit with the Hosmer–Lemeshow and likelihood ratio tests. Heteroskedasticity of residuals was assessed with the Breusch–Pagan/Cook–Weisberg test, and standardized robust errors were used to adjust for heteroskedasticity in the models as appropriate. All other assumptions were met. Data were analyzed and visualized using GraphPad Prism 8.2 (GraphPad software, San Diego, CA), and regression analyses were performed using Stata (version 14.2, Stata Corp, College Station, Texas, USA).

## Results

Forty-eight patients admitted to MICU and CTICU with median age of 61 (55–68), 56% (*n* = 27) males, and admission SOFA score of 8.1 ± 4.8 was enrolled in this study. Seven patients were removed due to missing ultrasound images at baseline due to assessor unavailable (*n* = 1) or images available could not be analyzed due to poor quality (*n* = 6). Demographic and clinical data of the forty-one patients included in the analyses are presented in Table 1. The time to first ultrasound measurement was median 1.1 days (IQR 0.77–1.4) after ICU admission. Paired ultrasound data were available for 35 patients on day 1 and day 7 of ICU admission, and 6 patients had missing images due to assessor unavailable (*n* = 2) or patient discharged prior to day 7 (*n* = 4), and thus, US data from ICU day 5 were utilized in analyses. Thirty-five patients participated in muscle strength, power and physical functional testing at hospital discharge. One patient’s time point was missed by researcher, and 5 patients died or transferred to inpatient hospice before discharge (Additional file 1: Fig. 1).

**Table 1:** Patient demographics and critical illness data

Parameter	(*n* = 41)
Age (years), median [IQR]	61 [55–68]
Male, *n* (%)	23 (56)
BMI (kg/m^2^), mean (SD)	29.6 (6.3)
Charlson comorbidity index, mean (SD)	5.5 (3.12)
Admitted to medical ICU, *n* (%)	30 (73)
Admitted to cardiothoracic ICU, *n* (%)	11 (27)
SOFA at ICU admission, mean (SD)	8.1 (4.8)
ICU LOS days, median [IQR]	8 [4]
Hospital LOS days, median [IQR]	11.2 [8–19]
MV, *n* (%)	30 (73)
MV, days, median [IQR]^a^	3.4 [1–7.7]
CRRT, *n* (%)	5 (12)
CRRT, days, median [IQR]^b^	9.8 [6.9–10.1]
ECMO, *n* (%)	2 (5)
Sedation, *n* (%)	24 (59)
Sedation, days, median [IQR]^c^	2 [1–3.25]
Inotropes and pressor, *n* (%)	25 (61)
Inotropes and pressor, days, median [IQR]^d^	4 [2–7]
Neuromuscular blocker, *n* (%)^e^	2 (5)
Time to first ultrasound measures, days, median [IQR]	1.1 (0.7–1.4)
Time to initial physical therapy session, days, mean (SD)	2.6 (1.84)
Time to initial occupational therapy session, days, mean (SD)	3.2 (2.71)
Number of rehabilitation visits for entire hospital stay, median, [IQR]	6 [4—9.25]
In-hospital mortality, *n* (%)	5 (12)

IQR = interquartile range; ICU = intensive care unit; BMI = body mass index; SOFA = sequential organ failure assessment; LOS = length of stay; ICU = intensive care unit; MV = mechanical ventilation; CRRT = continuous renal replacement therapy; ECMO = extra-corporeal membrane oxygenation;

^a^MV duration reported in days as median [IQR] for patients (*n* = 30) that required MV

^b^CRRT duration reported in days as median [IQR] for patients (*n* = 5) that required CRRT

^c^Duration of sedation reported for patients that received at least one sedative defined as number of days receiving at least one dosage

^d^Duration of inotrope and pressor for defined as the number of days a patient received at least one dosage

^e^2 patients received long-term NMB (8 and 23 days, respectively, in addition 23 patients received a one-time 50 mg doses of rocuronium for intubation or surgical procedure) (*n* = 23, 56%)

### Muscle ultrasound parameters (*n* = 41), *Fig. **1*

**Fig. 1:**
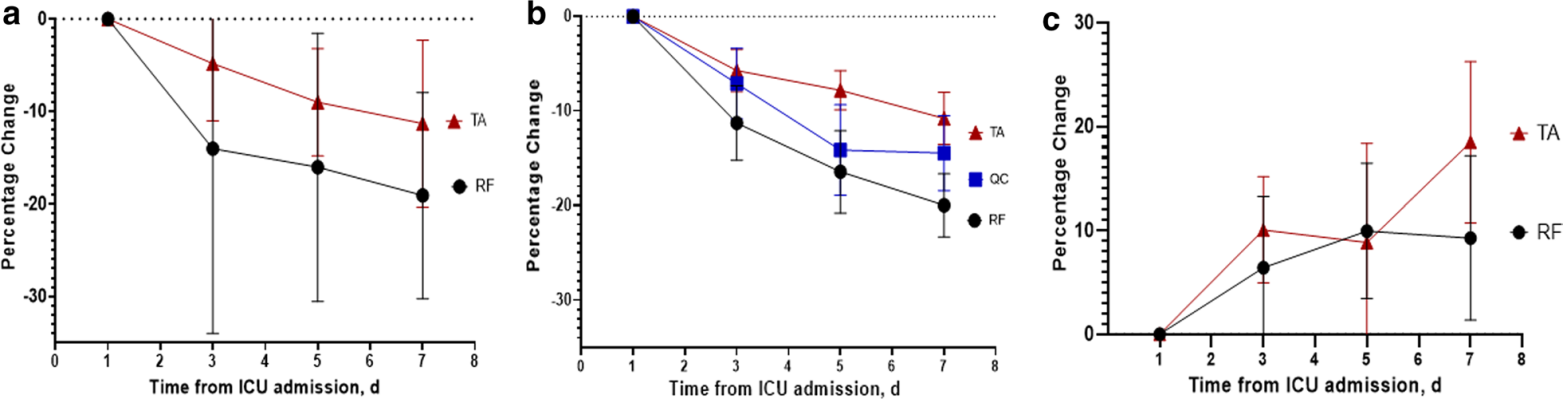
Change in rectus femoris and tibialis anterior muscle size and quality in first seven days of ICU stay. Panel (**a**) percent change of muscle layer thickness; panel (**b**) percent change of muscle cross-sectional area. (**c**) Percent change of echo intensity from day 1 to 7. d = days, RF = rectus femoris muscle; QC = quadricep complex muscles; TA = tibialis anterior muscle

#### mT

Rectus femoris mT at baseline was 0.98 ± 0.3 cm and decreased at median percent change of 20.1 (IQR 12 to 26%) from ICU day 1 to day 7, statistically significant change over time (*F* = 34.89, *p* = 0.0316). The quadriceps complex mT at baseline was 2.04 ± 0.71 cm and decreased at median percent change of 14.5 (IQR 7% to 24%) in the first seven days (*F* = 21.7, *p* = 0.003). Tibialis anterior muscle mT was 2.01 ± 0.36 cm at baseline and decreased at median percent change of 9.1 (IQR 5% to 12%) in the first seven days (*F* = 28.3, *p* < 0.001).

#### CSA

RF muscle CSA at baseline was 2.99 ± 0.99 cm^2^ and decreased at median percent change of 18.5% (IQR 11 to 23%) in the first seven days (*F* = 26.6, *p* = 0.0253). TA muscle CSA at baseline was 5.3 ± 0.89 cm^2^ and decreased at a median percent change of 8.1 (IQR 5 to 15%) in first seven days (*F* = 34.7, *p* < 0.001).

#### EI

Rectus femoris EI at baseline was 91 ± 24.9 and increased at a median percent change of 10.5% (IQR − 5 to 20%) in the first seven days (*F* = 3.28, *p* = 0.081). Tibialis anterior EI was 82.7 ± 21.2 at baseline and increased at median percent change of 15.4 (IQR 7 to 28%) within the first 7 days (*F* = 6.73, *p* = 0.002).

#### Muscle power

Twenty-six patients completed muscle power at ICU discharge with mean 8.0 ± 2.9 W for 2 lbs resistance and 44.8 ± 22.6 W for 10% of body weight test (Table 2). Muscle power increased from ICU to hospital discharge at a median percent change of 35% (IQR 15–55%) for 2 lbs resistance and 27% (IQR 7–48%) for 10% of BW resistance. Muscle power assessment at 2 lbs and 10% of BW were highly correlated, and therefore, only muscle power at 10% of BW was utilized in statistical analysis (Table 3).

**Table 2:** Muscle ultrasound, strength, power and physical function

Muscle parameter	Day 1	Day 7
Ultrasound parameters	*n* = 41	*n* = 41
TA mT (cm)	2.01 (0.36)	1.82 (0.31)
TA CSA (cmPP^2^PP)	5.28 (0.89)	4.71 (0.95)
TA EI (0–255)	82.7 (21.2)	96.7 (22.6)
RF mT (cm)	0.98 (0.3)	0.81 (0.27)
RF + VI mT (cm)	2.04 (0.71)	1.77 (0.62)
RF CSA (cmPP^2^PP)	2.99 (0.99)	2.47 (0.88)
RF EI (0–255)	90.7 (24.9)	99.1 (27.6)

	ICU discharge	Hospital discharge
Muscle power (W)	*n* = 26^a^	*n* = 33^b^
2 lbs	8.0 (2.89)	9.6 (3.5)
10% bodyweight	44.8 (22.6)	58.7 (30.6)
Muscle strength		
MRC-ss (0–60)	47.1 (7.3) (*n* = 31)^c^	51.4 (5.7) (*n* = 35)^e^
RF HHD force (kg)	16.9 (5.3) (*n* = 24)^d^	19.8 (6.9) (*n* = 31)^f^
RF HHD RFD (seconds)	3.8 (1.1) (*n* = 24)^d^	3.6 (1.1) (*n* = 31)^f^
TA HHD (kg)	14.5 (5.2) (*n* = 24)^d^	15.6 (5.4) (*n* = 31)^f^
TA HHD RFD (seconds)	3.9 (1.2) (*n* = 24)^d^	3.7 (1.2) (*n* = 31)^f^
Handgrip (kg)	18.2 (9.1) (*n* = 26)^a^	21.7 (10.0) (*n* = 32)^g^
Physical function		
SPPB	4.7 (3.9) (*n* = 26)^a^	5.9 (4.0) (*n* = 35)^e^
4-m gait speed (m/s)	0.49 (0.18) (*n* = 19)^h^	0.56 (0.27) (*n* = 31)^f^
5 × STS (seconds)	14.8 (5.6) (*n* = 13)^i^	18.9 (14.5) (*n* = 28)^j^
Balance	1.96 (1.4) (*n* = 19)^h^	2.3 (1.2) (*n* = 31)^f^
6 MWT distance (feet)	265 (182) (*n* = 26)^a^	455 (424) (*n* = 35)^e^
CFS	6.1 (1.5) (*n* = 36)	5.3 (1.7) (*n* = 36)

TA = tibialis anterior muscle; RF = rectus femoris muscle; CSA = cross-sectional area, mT = muscle layer thickness; EI = echointensity; MRC-ss = Medical Research Council-sum score; VI = vastus intermedius muscle; HHD = handheld dynamometer; RFD = rate of force development; SPPB = short performance physical battery; 5 × STS = five-times sit-to-stand test; 6 MWT = six-minute walk test; W = watts; CFS = clinical frailty scale

^a^Ten patients unable to complete test: 4 patients unable to follow commands/poor cognition; 3 patients had < 3/5 strength; 2 were missed by assessor; and 1 patient was unable to maintain oxygen saturations > 10% of baseline with simple movement in bed;

^b^Three unable to complete: 2 patients with < 3/5 strength and 1 patient unable to complete test: missed by assessor

^c^Five patients unable to complete: 4 patients unable to follow commands/poor cognition, 1 patient declined due to pain

^d^Twelve patients unable to complete test: patients reported in footnote b plus 2 patients fatigued after initial testing and physically were unable to perform HHD testing

^e^One patient declined due to pain

^f^Five patients unable to complete: 2 with < 3/5 strength, 2 deferred to pain/fatigue, 1 patient missed by assessor

^g^Four patients unable to complete: 2 with < 3/5 strength, 1 deferred to pain/fatigue, 1 patient missed by assessor

^h^Seventeen patients unable to complete test: patients reported in footnote b plus 7 patients unable to stand for balance or walk 4 m without physical assistance

^i^Twenty-three patients unable to complete test: patients reported in footnote b plus 13 patients unable to stand from chair in time allotted or without assistance

^j^Eight patients unable to complete: 2 with < 3/5 strength, 2 deferred to pain/fatigue, 1 patient missed by assessor, 3 patients could not perform without assistance

**Table 3:** Correlations between demographics, clinical data and muscle parameters measured in the ICU with physical function at hospital discharge

Variable	Muscle, Physical Function and Frailty assessed at Hospital Discharge, rs (*p* = 0.05)					
	Muscle power (10% BW)	5 × STS	ICU-AW	4-m gait speed	6 MWT	CFS
Age	− 0.543 (*p* = 0.001)	0.822 (*p* < 0.001)	0.269 (*p* = 0.118)	− 0.629 (*p* < 0.001)	− 0.596 (*p* < 0.001)	0.554 (*p* < 0.001)
BMI	0.096 (*p* = 0.597)	0.386 (*p* = 0.042)	− 0.285 (*p* = 0.097)	− 0.355 (*p* = 0.054)	− 0.210 (*p* = 0.219)	0.093 (*p* = 0.567)
CCI	− 0.006 (*p* = 0.973)	0.369 (*p* = 0.053)	0.137 (*p* = 0.431)	− 0.269 (*p* = 0.151)	− 0.359 (*p* = 0.032)	0.340 (*p* = 0.032)
SOFA	− 0.353 (*p* = 0.044)	− 0.352 (*p* = 0.07)	0.400 (*p* = 0.017)	0.262 (*p* = 0.162)	0.144 (*p* = 0.401)	− 0.219 (*p* = 0.174)
ICU LOS	0.090 (*p* = 0.618)	− 0.262 (*p* = 0.178)	0.348 (*p* = 0.041)	0.324 (*p* = 0.081)	0.028 (*p* = 0.872)	0.155 (*p* = 0.339)
Hospital LOS	0.109 (*p* = 0.545)	− 0.323 (*p* = 0.094)	0.440 (*p* = 0.008)	0.433 (*p* = 0.017)	0.061 (*p* = 0.722)	− 0.026 (*p* = 0.872)
RF mT (day 1)	0.248 (*p* = 0.160)	− 0.145 (*p* = 0.461)	− 0.308 (*p* = 0.072)	0.002 (*p* = 0.993)	0.059 (*p* = 0.732)	− 0.152 (*p* = 0.349)
Delta RF mT	0.112 (*p* = 0.534)	− 0.178 (*p* = 0.366)	− 0.272 (*p* = 0.114)	− 0.082 (*p* = 0.667)	− 0.079 (*p* = 0.646)	0.047 (*p* = 0.775)
RF CSA (day 1)	0.379 (*p* = 0.029)	− 0.230 (*p* = 0.248)	− 0.239 (*p* = 0.166)	0.131 (*p* = 0.491)	0.211 (*p* = 0.217)	− 0.239 (*p* = 0.138)
Delta RF CSA	− 0.159 (*p* = 0.375)	0.123 (*p* = 0.532)	− 0.181 (*p* = 0.297)	− 0.261 (*p* = 0.163)	− 0.105 (*p* = 0.541)	− 0.003 (*p* = 0.983)
RF EI (day 1)	− 0.480 (*p* = 0.005)	0.462 (*p* = 0.013)	0.337 (*p* = 0.048)	− 0.324 (*p* = 0.081)	− 0.295 (*p* = 0.080)	0.460 (*p* = 0.003)
Delta RF EI	0.150 (*p* = 0.406)	− 0.306 (*p* = 0.110)	− 0.214 (*p* = 0.218)	0.280 (*p* = 0.134)	0.190 (*p* = 0.268)	− 0.139 (*p* = 0.392)
PFIT-s*	0.670 (*p* < 0.001)	− 0.447 (*p* = 0.019)	− 0.640 (*p* < 0.001)	0.255 (*p* = 0.191)	0.648 (*p* < 0.001)	− 0.763 (*p* < 0.001)
MRC-ss*	0.333 (*p* = 0.090)	− 0.409 (*p* = 0.047)	− 0.626 (*p* < 0.001)	0.071 (*p* = 0.731)	0.429 (*p* = 0.018)	− 0.478 (*p* = 0.006)
Handgrip*	0.712 (*p* < 0.001)	− 0.416 (*p* = 0.001)	− 0.649 (*p* < 0.001)	0.167 (*p* = 0.447)	0.365 (*p* = 0.073)	− 0.489 (*p* = 0.011)
RF HHD*	0.837 (*p* < 0.001)	− 0.396 (*p* = 0.076)	− 0.597 (*p* = 0.003)	0.122(*p* = 0.589)	0.443 (*p* = 0.034)	− 0.551 (*p* = 0.005)
Muscle power (10% BW)*	0.819 (*p* < 0.001)	− 0.386 (*p* = 0.076)	− 0.541 (*p* = 0.005)	0.164 (*p* = 0.456)	0.384 (*p* = 0.058)	− 0.622 (*p* = 0.001)

Ultrasound images analyzed as baseline (day of ICU admission) and change in TA from day 1 to day 7 (delta). Data are displayed as Spearman Rho tests presented as correlation coefficient with *p* value

^*^Measured at ICU discharge, “Delta” represents the percentage change in muscle mT, CSA and EI from day 1 to day 7

CCI = Charlson comorbidity index; SOFA = sequential organ failure assessment; MRC-ss = Medical Research Council-sum score; RF = rectus femoris muscle; HHD = handheld dynamometry; SPPB = short performance physical battery; 5 × STS = five time sit-to-stand test; 6 MWT = six-minute walk test; mT = muscle layer thickness; CSA = cross-sectional; EI = echo-intensity; BW = body weight

#### ***Relationship between muscle size, quality, power and strength with physical function at hospital discharge (n*** = ***35)***

At ICU discharge 39% (12/31) met diagnosis for ICU-AW (Table 2). At hospital discharge the mean MRC-ss was 52.4 (5.7) with 25.7% (9/35) meeting criteria for ICU-AW. Handgrip strength was 21.7 ± 10 kg and RF muscle strength measured by HHD was 19.8 ± 6.9 kg with 3.6 ± 1.1 s to peak force production (Table 2). Patients scored an average 5.9 ± 4 on SPPB, with 0.56 ± 0.3 m/s gait speed and 18.9 ± 14 s to complete 5-times sit-to-stand test (Table 2). RF EI on day 1 of ICU admission was associated with muscle power (rs = − 0.48, *p* = 0.005), performance on 5 × STS (rs = 0.462, 0.013), ICU-AW (rs = 0.337, *p* = 0.048) and CFS score (0.460, 0.003) at hospital discharge (Table 3). Muscle power measured at ICU discharge was significantly related to ICU-AW and CFS at hospital discharge (Table 3). Muscle power measured at hospital discharge was also significantly related to age, SOFA at ICU admission, RF CSA, RF EI and measures of strength and function **(**Table 3**).**

#### Prediction modeling

Muscle power measured at ICU discharge, changes in rectus femoris CSA and EI from day one to seven, and sex predicted diagnosis of ICU-AW by < 48/60 on MRC-ss at hospital discharge in 25 patients with complete data. Muscle power and change in RF EI in first 7 days of ICU admission were the strongest predictors of ICU-AW (Table 4**, **area under curve = 0.912, Additional file 2: Fig. 2). Multivariate linear regression demonstrated that muscle power, age and ICU LOS are significant predictors of muscle 5 × STS performance at hospital discharge in 22 patients completing all measures (Table 4). Muscle power measured prior to ICU discharge was a strong independent predictor of sit-to-stand at hospital discharge.

**Table 4:** Multivariate logistic regression predicting ICU-AW at hospital discharge and multivariate linear regression predicting sit-to-stand performance at hospital discharge

Model *p* = 0.003 *R*^2^ = 0.51, VIF = 1	Odds ratio	SE	*z*	*P* >|*z*|	[95% CI]
*Dependent variable: diagnosis of ICU-AW at hospital discharge, n = 25*					
Power 10% BW in ICU	0.033	0.04	− 2.02	0.044	0.85, 0.99
Change in RF CSA days 1 to 7	< 0.001	0.0001	− 1.33	0.182	8.12e–13, 197.7
Change in RF EI days 1 to 7	4.40	0.0003	− 1.78	0.074	5.76e−12, 3.36
Male	0.53	1.25	1.56	0.787	0.005, 54.3

Model *p* = 0.04, *R*^2^ = 0.55, VIF 1.11	β-coefficient	SE	*t*	*P* >|*t*|	[95% CI]
*Dependent variable: performance on 5 × STS test at hospital discharge, n = 22*					
Power 10% BW in ICU	− 0.282	0.124	− 2.26	0.036	− 0.543, − 0.020
Age	0.534	0.173	3.09	0.006	0.171, 0.897
ICU LOS	− 0.091	0.033	− 2.76	0.013	− 0.161, − 0.0217

BW = bodyweight; RF = rectus femoris; CSA = cross-sectional area, EI = echo-intensity; ICU = intensive care unit; LOS = length of stay

## Discussion

The results of this study demonstrate that muscle ultrasound parameters, specifically RF EI, and lower extremity muscle power measured in the ICU are significant predictors of physical function at hospital discharge. Assessment of muscle quality by ultrasound and muscle power in the early course of critical illness, when combined with age and ICU LOS, may improve classification and prognostication of patients in the ICU at risk for weakness and physical dysfunction. Identifying the risk of physical impairments in critically ill patients upon admission or within the first few days in the ICU is important to improve clinical-decision making for therapeutic interventions. Timely assessment of skeletal muscle promotes an increased understanding of type and severity of muscle alterations, which may improve prognostication and lead to a more specific dosage of rehabilitation interventions and/or pharmacologic intervention to mitigate current or continued decline. Furthermore, muscle power is a novel concept that is rarely assessed in patients with and in those patients that have survived critical illness. The findings of this study suggest that muscle power should be incorporated in routine practice since power is a clinically important determinant of physical function.

Muscle power is not a current focus in critical care rehabilitation, but is a key component of functional mobility [21] and is important because it accounts for velocity (time and distance) to perform a task. Muscle power may present a novel therapeutic target with focus on an individualized training for patients with deficits. In older individuals, muscle power has been shown to decline earlier and at a steeper rate than muscle strength [52, 53], and therefore power training is a modality purported to mitigate the effects of sarcopenia [54]. Critical illness muscle wasting certainly has different underlying mechanisms of muscle atrophy when compared to mechanisms of age-related muscle mass loss. The concepts of muscle power training, however, may be beneficial in both populations. Additionally, more than half of ICU admissions in the USA are older individuals (> 65 years of age) [55] and thus suggest muscle power is an important construct in muscle and physical dysfunction for those critically ill. The ability to generate force, quickly to overcome gravity to stand from seated position requires lower-extremity muscle power [56]. Previous data suggest that older patients and those with longer mechanical ventilation will have delayed time to achieve independence with sit-to-stand transfer [57]. Thus, 5 × STS was selected as the primary physical function outcome of interest since it has strong construct validity with muscle strength and power, an important measure of functional mobility [58, 59]. Changes in muscle power may be explained by a selective decrease in type-II muscle fibers, which are most important for power production. Data from muscle biopsies demonstrate that type-II fibers have smaller CSA and potentially decrease at more predominant rate than type-I fibers in patients requiring mechanical ventilation [60, 61]. Data from muscle power assessment in this study had moderate to strong correlations with rectus femoris muscle size, muscle EI, strength and physical function. Rectus femoris muscle has a high composition of type IIA and IIX muscle fibers [62] which supports the relationship between muscle power and rectus femoris muscle size and quality in this study. Muscle power increased from ICU to hospital discharge, which may suggest time points in the ICU, may be influenced by limited voluntary muscle contraction when patients are acutely ill. Muscle power measured at hospital discharge in this cohort was significantly reduced compared to previously reported data from healthy, age-matched controls (reductions up to 47%) [41]. Muscle power should be explored in future studies to understand long-term recovery.

Results of the current study confirm the rapid and significant deterioration in skeletal muscle size and quality in patients admitted to the ICU for critical illness that have been reported in prior published work [1, 25, 63, 64]. We demonstrated decrease in RF muscle CSA of 19% in first week of critical illness, slightly higher than prior data ranging from decreases of 12.5% to 17% [1, 25, 65]. It should be noted that baseline RF muscle size (mT and CSA) was lower when compared to previous studies [25, 64]. This may be explained by differences in landmarking, variability in sonographer compression technique and, more likely, differences in study populations. Specifically, the inclusion of patients in the cardiothoracic ICU with heart or lung failure with potential for chronic wasting and frailty may explain part of the differences in baseline rectus femoris muscle size. Differences in techniques and heterogeneous populations confirm the need to develop standardized approaches when performing muscle ultrasound in the ICU [27]. EI, a marker of muscle quality [66], increased across these same time points by 10.5%, which is similar to prior published data (+ 9.6%) [25]. These changes are purported to be clinically meaningful deteriorations in the muscle structure potentially related to myofiber necrosis [66, 67].

Muscle ultrasound is a non-invasive and relatively inexpensive tool that can be implemented early during critical illness to potentially expedite classification of muscle mass and quality. Early diagnosis and classification of patients at risk for physical impairments may improve outcomes by promoting earlier allocation or greater intensity (number of visits) of physical rehabilitation. Current diagnosis of ICU-AW is typically delayed until the patient can volitionally engage in the MRC-ss [35, 68]. Therefore, ultrasound used early in the time course of critical illness when patients are not yet able to volitionally engage may improve assessment of muscle dysfunction. Data from this study demonstrate that deterioration in rectus femoris muscle quality is moderately and significantly correlated with ICU-AW, physical function and clinical frailty scale at hospital discharge. Therefore, this study provides preliminary data to suggest that quantification of muscle quality with ultrasound imaging can improve classification of patients at risk for ICU-AW and physical impairments. The findings may also suggest that muscle size may not be the best predictor of outcomes, specifically ICU-AW. Muscle mass or size has previously been shown not to correlate with muscle strength [69, 70], potentially demonstrating that atrophy may not be the primary culprit of ICU-AW. These data, interpreted with caution, may support that deteriorations in muscle quality and muscle power may partially explain development of ICU-AW.

The primary limitation of this study is the small sample size limiting the strength of correlations and the strength of the modeling or prediction analyses. Multivariate logistic and linear regression were performed as exploratory analyses and should be interpreted with caution due to the study being under-powered. The study was not powered to conduct group analyses and such we focused on the descriptive data and correlations. Additional exploratory analyses were not performed in this study as the primary aim was focused on early muscle assessment to predict physical function at hospital discharge. A secondary limitation is some missing data due to assessor availability or the patient unable to complete tests due to pain, lack of cognitive function or change in care to hospice or comfort care. Finally, research conducted in the ICU is limited due to timing; it is likely that patients have suffered changes in muscle and physical function long before admission to the ICU which makes establishing a baseline nearly impossible.

## Conclusion

In this study we showed that changes in muscle quality and power assessed in the ICU are significantly related to physical function in patients with critical illness. Muscle power could be an important clinical measure to be considered in the assessment of patients with and those patients that have survived critical illness.

## Supplementary information

**Additional file 1.** Flow diagram of patients screening, enrolled and participating.^pre-existing neuromuscular, neurologic, or orthopedic condition that would prevent participation in functional tests; EtOH = alcoholic abuse; IVDU = intra-venous drug usage/abuse; BMI = body mass index; PLOF = prior level of function; LAR =legally authorized representative; US = ultrasound

**Additional file 2.** Receiver operator curve of multivariate logistic regression predicting ICU-AW at hospital discharge

## Data Availability

The minimal data are included in this published article. The datasets used and/or analyzed during the current study are available from the corresponding author on reasonable request.

## References

[1] Puthucheary ZA, Rawal J, McPhail M (2013). Acute skeletal muscle wasting in critical illness. JAMA.

[2] Fan E, Dowdy DW, Colantuoni E (2014). Physical complications in acute lung injury survivors: a two-year longitudinal prospective study. Crit Care Med.

[3] Sharshar T, Bastuji-Garin S, Stevens RD (2009). Presence and severity of intensive care unit-acquired paresis at time of awakening are associated with increased intensive care unit and hospital mortality. Crit Care Med.

[4] Herridge MS, Tansey CM, Matte A (2011). Functional disability 5 years after acute respiratory distress syndrome. New Engl J Med.

[5] Hough CL, Herridge MS (2012). Long-term outcome after acute lung injury. Curr Opin Crit Care.

[6] Griffith DM, Salisbury LG, Lee RJ (2018). Determinants of health-related quality of life after ICU: importance of patient demographics, previous comorbidity, and severity of illness. Crit Care Med.

[7] Cuthbertson BH, Roughton S, Jenkinson D, Maclennan G, Vale L (2010). Quality of life in the five years after intensive care: a cohort study. Critical Care (Lond, Engl).

[8] Schweickert WD, Pohlman MC, Pohlman AS (2009). Early physical and occupational therapy in mechanically ventilated, critically ill patients: a randomised controlled trial. Lancet (London, England).

[9] Morris PE, Griffin L, Berry M (2011). Receiving early mobility during an intensive care unit admission is a predictor of improved outcomes in acute respiratory failure. Am J Med Sci.

[10] Schaller SJ, Anstey M, Blobner M (2016). Early, goal-directed mobilisation in the surgical intensive care unit: a randomised controlled trial. Lancet (London, England).

[11] Tipping CJ, Harrold M, Holland A, Romero L, Nisbet T, Hodgson CL (2017). The effects of active mobilisation and rehabilitation in ICU on mortality and function: a systematic review. Intensive Care Med.

[12] Balas MC, Devlin JW, Verceles AC, Morris P, Ely EW (2016). Adapting the ABCDEF bundle to meet the needs of patients requiring prolonged mechanical ventilation in the long-term acute care hospital setting: historical perspectives and practical implications. Semin Respirat Crit Care Med.

[13] Devlin JW, Skrobik Y, Gelinas C (2018). Clinical practice guidelines for the prevention and management of pain, agitation/sedation, delirium, immobility, and sleep disruption in adult patients in the ICU. Crit Care Med.

[14] Wright SE, Thomas K, Watson G (2018). Intensive versus standard physical rehabilitation therapy in the critically ill (EPICC): a multicentre, parallel-group, randomised controlled trial. Thorax.

[15] Cuthbertson BH, Rattray J, Campbell MK (2009). The PRaCTICaL study of nurse led, intensive care follow-up programmes for improving long term outcomes from critical illness: a pragmatic randomised controlled trial. BMJ (Clin Res ed).

[16] Denehy L, Skinner EH, Edbrooke L (2013). Exercise rehabilitation for patients with critical illness: a randomized controlled trial with 12 months of follow-up. Crit Care (Lond, Engl).

[17] Moss M, Nordon-Craft A, Malone D (2016). A randomized trial of an intensive physical therapy program for patients with acute respiratory failure. Am J Respir Crit Care Med.

[18] Waldauf P, Jiroutková K, Krajčová A, Puthucheary Z, Duška F (2020). Effects of rehabilitation interventions on clinical outcomes in critically ill patients: systematic review and meta-analysis of randomized controlled trials. Crit Care Med.

[19] Iwashyna TJ, Burke JF, Sussman JB, Prescott HC, Hayward RA, Angus DC (2015). Implications of heterogeneity of treatment effect for reporting and analysis of randomized trials in critical care. Am J Respir Crit Care Med.

[20] Bean JF, Kiely DK, Herman S (2002). The relationship between leg power and physical performance in mobility-limited older people. J Am Geriatr Soc.

[21] Reid KF, Fielding RA (2012). Skeletal muscle power: a critical determinant of physical functioning in older adults. Exerc Sport Sci Rev.

[22] Foldvari M, Clark M, Laviolette LC (2000). Association of muscle power with functional status in community-dwelling elderly women. J Gerontol Ser A.

[23] Appleton RT, Kinsella J, Quasim T (2015). The incidence of intensive care unit-acquired weakness syndromes: a systematic review. J Intensive Care Soc.

[24] Witteveen E, Sommers J, Wieske L (2017). Diagnostic accuracy of quantitative neuromuscular ultrasound for the diagnosis of intensive care unit-acquired weakness: a cross-sectional observational study. Ann Intens Care.

[25] Parry SM, El-Ansary D, Cartwright MS (2015). Ultrasonography in the intensive care setting can be used to detect changes in the quality and quantity of muscle and is related to muscle strength and function. J Crit Care.

[26] Hadda V, Kumar R, Khilnani GC (2018). Trends of loss of peripheral muscle thickness on ultrasonography and its relationship with outcomes among patients with sepsis. J Intensive Care.

[27] Mourtzakis M, Parry S, Connolly B, Puthucheary Z (2017). Skeletal muscle ultrasound in critical care: a tool in need of translation. Ann Am Thoracic Soc.

[28] Mayer K, Boustany H, Cassity E, et al. ICU recovery clinic attendance, attrition and patient outcomes: the impact of severity of illness, gender and rurality. *Critical Care Explorations.* 2020; In Press. 10.1097/CCE.0000000000000206 PMC7523871 33063022

[29] Mayer KP, Dhar S, Cassity E, et al. Interrater reliability of muscle ultrasonography image acquisition by physical therapists in patients who have or who survived critical illness. *Physical therapy.* 2020. 10.1093/ptj/pzaa068 32302406

[30] Seymour JM, Ward K, Sidhu PS (2009). Ultrasound measurement of rectus femoris cross-sectional area and the relationship with quadriceps strength in COPD. Thorax.

[31] Sarwal A, Parry SM, Berry MJ (2015). Interobserver reliability of quantitative muscle sonographic analysis in the critically Ill population. J Ultrasound Med.

[32] Connolly B, MacBean V, Crowley C (2015). Ultrasound for the assessment of peripheral skeletal muscle architecture in critical illness: a systematic review. Crit Care Med.

[33] Mourtzakis M, Wischmeyer P (2014). Bedside ultrasound measurement of skeletal muscle. Curr Opin Clin Nutrit Metab Care.

[34] De Jonghe B, Sharshar T, Lefaucheur JP (2002). Paresis acquired in the intensive care unit: a prospective multicenter study. JAMA.

[35] Connolly BA, Jones GD, Curtis AA (2013). Clinical predictive value of manual muscle strength testing during critical illness: an observational cohort study. Crit Care (Lond, Engl).

[36] Hough CL, Lieu BK, Caldwell ES (2011). Manual muscle strength testing of critically ill patients: feasibility and interobserver agreement. Crit Care.

[37] Parry SM, Berney S, Granger CL (2015). A new two-tier strength assessment approach to the diagnosis of weakness in intensive care: an observational study. Crit Care (Lond, Engl).

[38] Stark T, Walker B, Phillips JK, Fejer R, Beck R (2011). Hand-held dynamometry correlation with the gold standard isokinetic dynamometry: a systematic review. PM & R.

[39] Bohannon RW (1986). Test-retest reliability of hand-held dynamometry during a single session of strength assessment. Phys Ther.

[40] Baldwin CE, Paratz JD, Bersten AD (2013). Muscle strength assessment in critically ill patients with handheld dynamometry: an investigation of reliability, minimal detectable change, and time to peak force generation. J Crit Care.

[41] Mayer K, Evans C, Welle M, et al. Muscle power is related to physical function in patients surviving acute respiratory failure: a prospective observational study. *American Journal of Medical Science.* 2020; Accepted, In Press. 10.1016/j.amjms.2020.09.018 33189316

[42] Melo TAD, Duarte ACM, Bezerra TS, França F, Soares NS, Brito D (2019). The Five Times Sit-to-Stand Test: safety and reliability with older intensive care unit patients at discharge. Revista Brasileira de terapia intensiva..

[43] Parry SM, Denehy L, Beach LJ, Berney S, Williamson HC, Granger CL (2015). Functional outcomes in ICU - what should we be using? An observational study. Crit Care (Lond, Engl).

[44] Chan KS, Aronson Friedman L, Dinglas VD (2016). Evaluating physical outcomes in acute respiratory distress syndrome survivors: validity, responsiveness, and minimal important difference of 4-meter gait speed test. Crit Care Med.

[45] Needham DM, Sepulveda KA, Dinglas VD (2017). core outcome measures for clinical research in acute respiratory failure survivors. An international modified delphi consensus study. Am J Respirat Crit Care Med..

[46] ATS statement: guidelines for the six-minute walk test. *American journal of respiratory and critical care medicine.* 2002;166(1):111–117. 10.1164/ajrccm.166.1.at1102 12091180

[47] Juma S, Taabazuing M-M, Montero-Odasso M (2016). Clinical frailty scale in an acute medicine unit: a simple tool that predicts length of stay. Can Geriatr J CGJ.

[48] Hodgson CL, Stiller K, Needham DM (2014). Expert consensus and recommendations on safety criteria for active mobilization of mechanically ventilated critically ill adults. Crit Care (Lond Engl).

[49] Parry SM, Granger CL, Berney S (2015). Assessment of impairment and activity limitations in the critically ill: a systematic review of measurement instruments and their clinimetric properties. Intensive Care Med.

[50] Denehy L, de Morton NA, Skinner EH (2013). A physical function test for use in the intensive care unit: validity, responsiveness, and predictive utility of the physical function ICU test (scored). Phys Ther.

[51] Taylor BE, McClave SA, Martindale RG (2016). Guidelines for the provision and assessment of nutrition support therapy in the adult critically ill patient: society of critical care medicine (sccm) and american society for parenteral and enteral nutrition (ASPEN). Crit Care Med..

[52] Skelton DA, Greig CA, Davies JM, Young A (1994). Strength, power and related functional ability of healthy people aged 65–89 years. Age Ageing.

[53] McKinnon NB, Connelly DM, Rice CL, Hunter SW, Doherty TJ (2017). Neuromuscular contributions to the age-related reduction in muscle power: Mechanisms and potential role of high velocity power training. Ageing Res Rev.

[54] Bottaro M, Machado SN, Nogueira W, Scales R, Veloso J (2007). Effect of high versus low-velocity resistance training on muscular fitness and functional performance in older men. Eur J Appl Physiol.

[55] Milbrandt EB, Eldadah B, Nayfield S, Hadley E, Angus DC (2010). Toward an integrated research agenda for critical illness in aging. Am J Respir Crit Care Med.

[56] Millor N, Cadore EL, Gómez M (2020). High density muscle size and muscle power are associated with both gait and sit-to-stand kinematic parameters in frail nonagenarians. J Biomech.

[57] Thomas S, Burridge JH, Pohl M, Oehmichen F, Mehrholz J (2016). Recovery of sit-to-stand function in patients with intensive-care-unit-acquired muscle weakness: results from the general weakness syndrome therapy cohort study. J Rehabil Med.

[58] Bohannon RW, Bubela DJ, Magasi SR, Wang YC, Gershon RC (2010). Sit-to-stand test: Performance and determinants across the age-span. Isokinet Exerc Sci.

[59] Jones SE, Kon SS, Canavan JL (2013). The five-repetition sit-to-stand test as a functional outcome measure in COPD. Thorax.

[60] Wollersheim T, Woehlecke J, Krebs M (2014). Dynamics of myosin degradation in intensive care unit-acquired weakness during severe critical illness. Intensive Care Med.

[61] Bierbrauer J, Koch S, Olbricht C (2012). Early type II fiber atrophy in intensive care unit patients with nonexcitable muscle membrane. Crit Care Med.

[62] Methenitis S, Karandreas N, Spengos K, Zaras N, Stasinaki AN, Terzis G (2016). Muscle fiber conduction velocity, muscle fiber composition, and power performance. Med Sci Sports Exerc.

[63] Gruther W, Benesch T, Zorn C (2008). Muscle wasting in intensive care patients: ultrasound observation of the M. quadriceps femoris muscle layer. J Rehabilit Med..

[64] Cartwright MS, Kwayisi G, Griffin LP (2013). Quantitative neuromuscular ultrasound in the intensive care unit. Muscle Nerve.

[65] McNelly AS, Bear DE, Connolly BA (2020). Effect of intermittent or continuous feed on muscle wasting in critical illness: a phase 2 clinical trial. Chest.

[66] Puthucheary ZA, Phadke R, Rawal J (2015). Qualitative ultrasound in acute critical illness muscle wasting. Crit Care Med.

[67] Reimers K, Reimers CD, Wagner S, Paetzke I, Pongratz DE (1993). Skeletal muscle sonography: a correlative study of echogenicity and morphology. J Ultrasound Med.

[68] Hermans G, Clerckx B, Vanhullebusch T (2012). Interobserver agreement of medical research council sum-score and handgrip strength in the intensive care unit. Muscle Nerve.

[69] Wollersheim T, Grunow JJ, Carbon NM (2019). Muscle wasting and function after muscle activation and early protocol-based physiotherapy: an explorative trial. Journal of cachexia, sarcopenia and muscle.

[70] Dos Santos C, Hussain SN, Mathur S (2016). Mechanisms of chronic muscle wasting and dysfunction after an intensive care unit stay. A pilot study. Am J Respirat Crit Care Med..

