# Supplementary figures and images for: Acute skeletal muscle wasting and dysfunction predict physical disability at hospital discharge in patients with critical illness
Source: Crit Care. 2020 Nov 4;24:637. doi: 10.1186/s13054-020-03355-x (PMC7640401; doi:10.1186/s13054-020-03355-x)

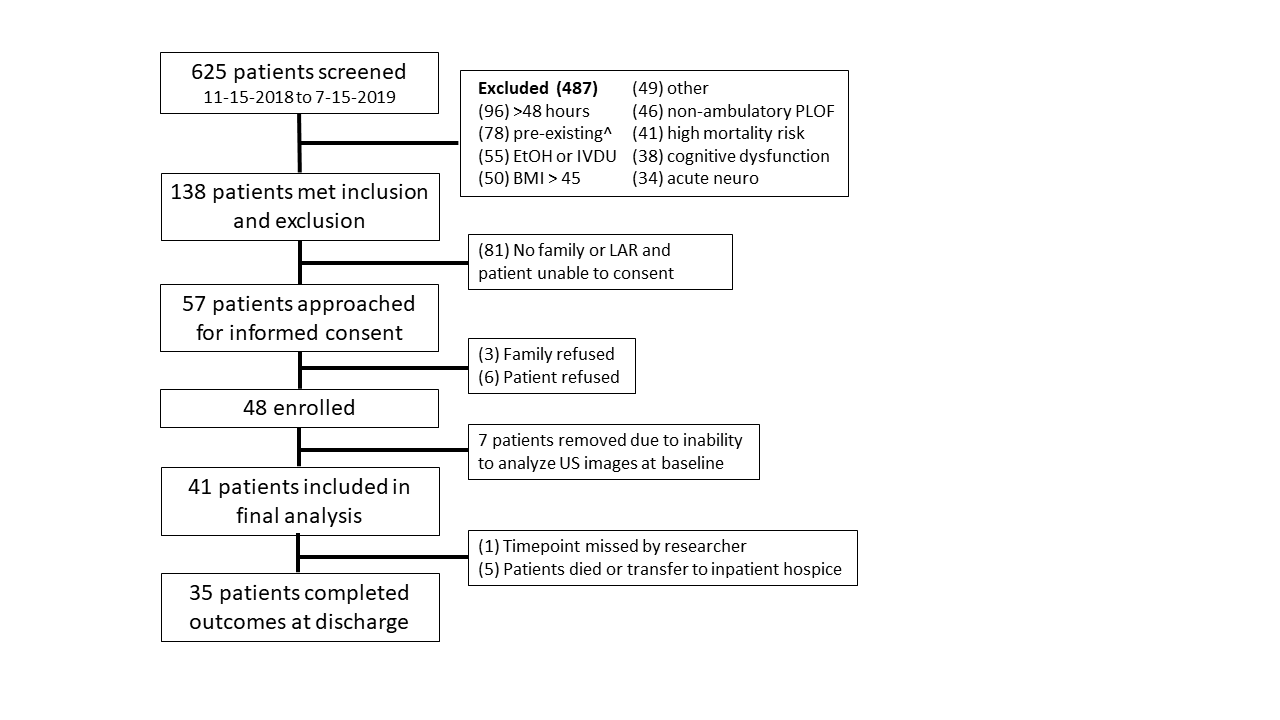

Supplement: Supplementary file 1 — Additional file 1. Flow diagram of patients screening, enrolled and participating.^pre-existing neuromuscular, neurologic, or orthopedic condition that would prevent participation in functional tests; EtOH = alcoholic abuse; IVDU = intra-venous drug usage/abuse; BMI = body mass index; PLOF = prior level of function; LAR =legally authorized representative; US = ultrasound [file 13054_2020_3355_MOESM1_ESM.tif]

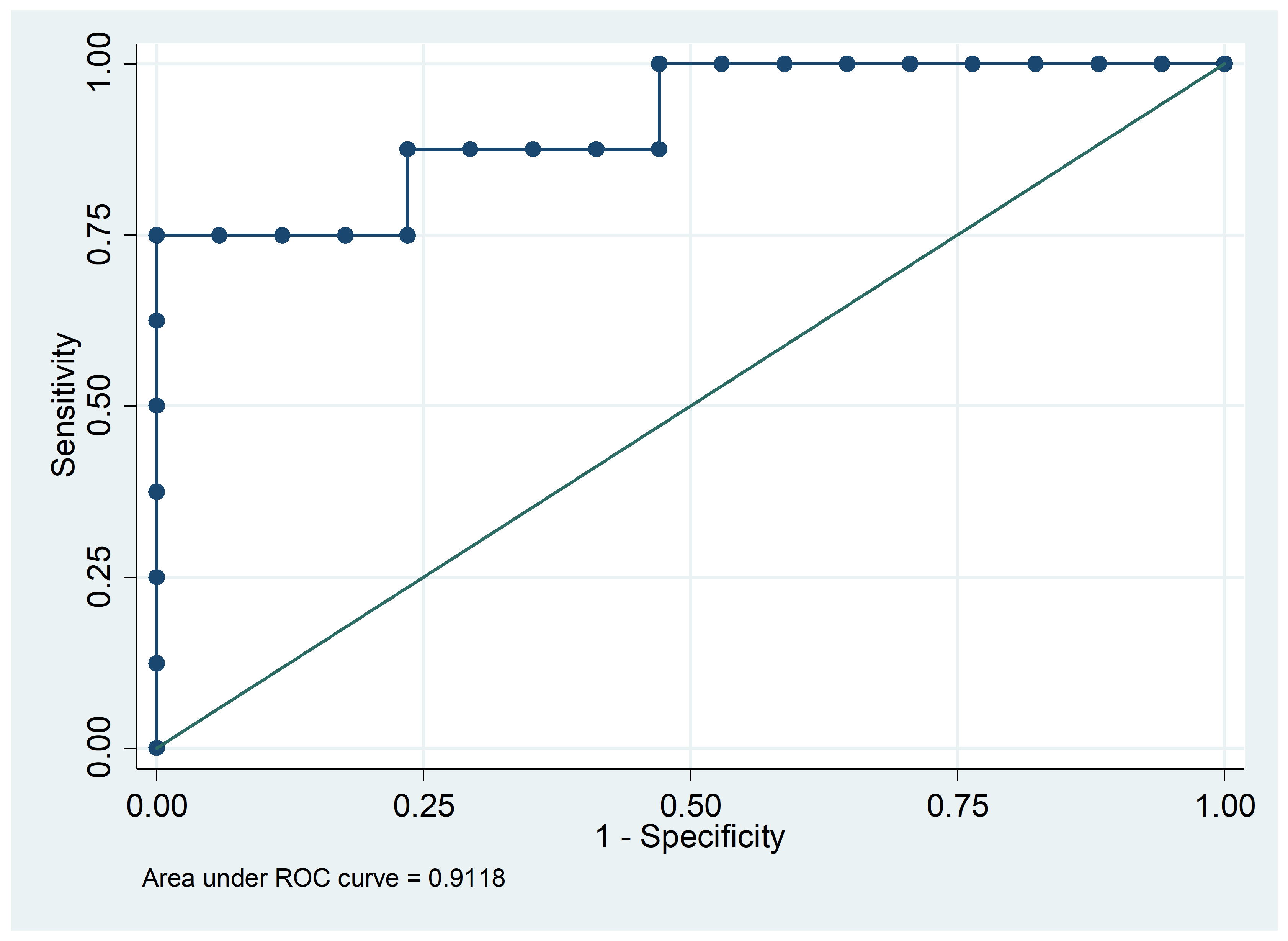

Supplement: Supplementary file 2 — Additional file 2. Receiver operator curve of multivariate logistic regression predicting ICU-AW at hospital discharge [file 13054_2020_3355_MOESM2_ESM.tif]
